# Cortisol and α-Amylase Secretion Patterns between and within Depressed and Non-Depressed Individuals

**DOI:** 10.1371/journal.pone.0131002

**Published:** 2015-07-06

**Authors:** Sanne H. Booij, Elisabeth H. Bos, Mara E. J. Bouwmans, Martijn van Faassen, Ido P. Kema, Albertine J. Oldehinkel, Peter de Jonge

**Affiliations:** 1 Interdisciplinary Center Psychopathology and Emotion regulation, Department of Psychiatry, University of Groningen, University Medical Center Groningen, Groningen, The Netherlands; 2 Center for Integrative Psychiatry, Lentis, Groningen, The Netherlands; 3 Department of Laboratory Medicine, University Medical Center Groningen, Groningen, The Netherlands; Brock University, CANADA

## Abstract

**Objectives:**

Associations between biological stress markers and depression are inconsistent across studies. We assessed whether inter- and intra-individual variability explain these inconsistencies.

**Methods:**

Pair-matched depressed and non-depressed participants (N = 30) collected saliva thrice a day for 30 days, resulting in 90 measurements per individual. The relationships between measures of stress-system function and depression were examined at the group level by means of mixed model analyses, and at the individual level by means of pair-matched comparisons. The analyses were repeated after adjusting for time-varying lifestyle factors by means of time-series regression analyses.

**Results:**

Cortisol and α-amylase levels were higher, the α-amylase/cortisol ratio larger, and the daily cortisol slope steeper in the depressed compared to the non-depressed group. Adjusting for lifestyle factors and antidepressant use reduced the associations under study. In 40%–60% of the matched comparisons, depressed individuals had higher cortisol and α-amylase levels, a larger α-amylase/cortisol ratio, and a steeper daily slope than their non-depressed match, regardless of adjustment.

**Conclusions:**

Our group-level findings were mostly in line with the literature but generalization to individuals appeared troublesome. Findings of studies on this topic should be interpreted with care, because in clinical practice the focus is on individuals instead of groups.

## Introduction

Cortisol and α-amylase are both markers of the human stress system, but reflect distinct components of it. Cortisol is released from the adrenal gland as the end product of the hypothalamic-pituitary-adrenal (HPA) axis, a relatively slow system that adapts the body to stress conditions by mobilizing energy and inhibiting non-emergency processes, such as sleep, sexual activity and growth [1]. Alpha-amylase is an enzyme that hydrolyses starch in the oral cavity; its secretion is under autonomic regulation and is highly sensitive to stress-related changes [2]. The autonomic nervous system (ANS) is a fast system that enables the body to cope with a stressor directly by effecting the cardiovascular and respiratory system and releasing catecholamines [3]. Both cortisol and α-amylase have been studied in relation to mood problems [2].

Over the past years, the functioning of the HPA axis and its end-product cortisol in depressed patients has received much attention [4]. Salivary cortisol levels have been studied at various moments of the day, in naturalistic as well as experimental settings [5]. In a meta-analysis, Stetler and Miller summarized four decades of research into HPA axis activation and depression, and concluded that there seems to be a tendency for increased HPA axis activation in depressed samples [5]. They also noted that results varied considerably across studies, and that several studies showed decreased instead of increased activation of the HPA axis. Depression has also been associated with a flatter diurnal cortisol rhythm, but this has been investigated less often, and the available evidence is not very consistent [6]. The few studies that examined salivary α-amylase levels in relation to depressive symptoms show a tendency towards increased α-amylase levels in depressed versus non-depressed groups, but these results are mixed as well [2].

In 2002, Bauer and colleagues argued that an adaptive stress response should involve interaction of the HPA axis and the ANS in a coordinate manner [7]. An imbalance of the two systems might therefore result in several health problems [2]. A recent study found that the ratio of stress-induced salivary α-amylase over cortisol was more strongly related to depressive symptoms and to several indices of chronic stress than either of them alone. More specifically, relatively high α-amylase combined with relatively low cortisol levels predicted poor outcomes [8]. However, another study found no interaction between cortisol and α-amylase in the prediction of depressive symptoms [9]. Hence, the interplay of these two components of the stress system deserves more scrutiny.

The inconsistencies in the present literature may, at least partly, be the result of interference by within-subject variability. Salivary cortisol and α-amylase levels vary considerably over time within individuals, because they are influenced by many fluctuating factors, among which circadian phase, ultradian phase, time of waking up, food intake, substance use, physical activity, daily hassles, and social interactions [10–12]. To adequately capture variation in cortisol and α-amylase at the group level, intra-individual variation should be dealt with as well [13]. This requires multiple measurements per individual. The exact number of measurements needed depends on the homogeneity of the population sample, the timing and interval of the measurements, and whether important confounding factors are taken into account. Hruschka et al. calculated, for two example studies, with very different sample sizes and sampling intervals, that 70 and 88 measurements would have been necessary to estimate between-individual differences in diurnal slope with moderate reliability, while 12 and 15 samples were taken, respectively [13]. Up till now, only a few studies have sampled cortisol more often, that is, 50–60 times [14,15]. In the present study, we measured cortisol and α-amylase three times a day over 30 days (T = 90) in 30 pair-matched depressed and non-depressed individuals. Such lengthy research is only feasible if conducted in the participants’ own environment. This has the added benefit that generalization to real life is more sensible than in laboratory studies [16,17].

Inconsistencies in the literature may also be caused by individual heterogeneity. Group studies into the relationship between cortisol and depression generally aim to increase knowledge about the etiology and treatment of *individuals* with depression. But what is found to be true at the group level (i.e., on average) is not necessarily true for individuals [18]. The present study focused on multiple repeated measurements in pair-matched individuals with and without depression. This allowed us to determine how well group results generalized to the level of individuals, and to zoom in on individual time series to identify potential sources of heterogeneity. In sum, this study complements the available literature in a unique way by combining a nomothetic and idiographic approach to assess whether depression is related to cortisol and α-amylase secretion patterns.

## Materials and Methods

### Participants

The data were collected as part of the ‘Mood and movement in daily life’ (MOOVD) study, which was set up to investigate the dynamic relationship between (physical) activity and mood in daily life, and the role of several biomarkers therein. Participants (age 20–50 years) were intensively monitored in their natural environments for 30 days, by means of electronic diaries, actigraphy, and saliva sampling, resulting in a total of 90 measurements per individual. Of the 62 participants who started the study, 4 participants dropped out early. Another 4 participants completed the study but did not have enough valid physical activity or diary measurements (T<60), leaving 54 participants for further study. Participants with and without a depressive disorder were pair-matched on gender, smoking, age and BMI. For the first 15 matched pairs, cortisol and α-amylase samples were analyzed. These participants make up the present study sample. They did not differ significantly from the remaining participants on BDI score, gender, age, BMI and smoking status (p>0.05). All matched pairs had the same gender and smoking status, while age and BMI differed on average 3.1 years (SD = 2.4) and 3.4 kg/l^2^ (SD = 2.6). Pairs are numbered 1–15, preceded by a D (indicating depression) or an N (no depression).

The depressed participants were recruited from patients of the Psychiatry Department of the University Medical Centre Groningen (UMCG) and the Centre for Integrative Psychiatry (CIP) in Groningen, The Netherlands. The non-depressed participants were recruited from the general population by means of posters and advertisements in local newspapers. As a first step, all participants were screened by means of the Beck Depression Inventory (BDI-II) [19] and a general health questionnaire. The BDI score had to be higher than 14 to be included in the depression group, and lower than 9 to be included in the non-depressed group. Eligible individuals were invited to an introduction session, including the Composite International Diagnostic Interview [20], several questionnaires, and an explanation of the use of equipment and the daily diary. Depressed individuals were included if they had a DSM-IV diagnosis of Major Depressive Disorder (current episode or in remission for less than 8 weeks). Non-depressed individuals were included if they were free of mood disorders at the moment of inclusion. Individuals with a current or recent (within the last two years) psychotic or bipolar disorder, a chronic somatic illness, or medication use known to influence the functioning of the HPA axis or the ANS (e.g. corticosteroids, beta-blockers) were excluded. Antidepressant medication was allowed. Other reasons for exclusion were pregnancy and significant hearing or visual impairments. At the end of the study, the participants were debriefed and filled out several questionnaires. We offered a minimum of €60 for participation, and bonus fees according to the number of completed daily questionnaires. Additionally, the participants received a personal report on their daily mood and activity patterns within 3 months after the end of the study.

### Ethics statement

All participants gave written consent, and the MOOVD study design was approved by the Medical Ethical Committee of the University Medical Center Groningen. The study was conducted in accordance with the Declaration of Helsinki.

### Ambulatory sampling

The participants completed questionnaires on an electronic diary, the PsyMate^22^ (PsyMate BV, Maastricht, The Netherlands) [21] for a total of 32 days, whereof the first two days served to get familiar with the device. The electronic diary questionnaire contained 60 items on mood, cognition, and daily activities. The PsyMate was programmed to generate beeps at three predetermined moments a day at equidistant time points: in the morning, six hours later in the afternoon, and again six hours later in the evening. To capture most of the participants’ daily life without intruding with their sleep habits, the beeps were planned preferably at the end of the morning, afternoon and evening. The exact time points depended on the participants’ sleep-wake schedule, as assessed by the Munich Chronotype Questionnaire (MCTQ) [22] and a question regarding the time they would go to bed if they were to go to bed ‘on time’. This is convenient for the subjects, and it also reduces differences between individuals due to circadian phase; studies into circadian rhythmicity have shown that there are individual differences in a person’s natural sleep-wake rhythm [22], and the accompanied (phase-locked) hormonal rhythms, such as that of cortisol and melatonin [23,24]. The depressed and non-depressed group did not differ with regard to the time they filled out the daily questionnaires. After every alarm beep, the participants were asked to fill out the electronic diary. They were instructed to do so immediately after the beep, but a time window of 1 hour was allowed when this was not possible. Saliva was usually collected while completing the diary, by means of a synthetic collection device, the Salivette. As recommended elsewhere [25], the participants were asked to keep the Salivette in their mouth for at least 2 minutes and to refrain from chewing. They were not allowed to eat or drink anything except water, nor to smoke or brush their teeth within 30 minutes before saliva sampling. As a reminder of these restrictions, an extra alarm beep and a text message were presented 30 minutes before every diary beep. Throughout the study, the participants wore the ActiCal (Respironics, Bend, OR, USA), an omnidirectional water-resistant accelerometer with the size of a watch, around the wrist of the non-writing arm. They were instructed never to remove the ActiCal except when entering a sauna or sunbed (high temperatures). The ActiCal recorded data over 1-min intervals. For more details about the ActiCal, see elsewhere [26].

### Salivary cortisol and α-amylase

The participants stored their saliva samples in a refrigerator as soon as possible. They reported samples that had been out of a refrigerator for more than 4 hours (2.4%) in a logbook. Research staff collected the samples every week, and brought them to the special chemistry Lab of the UMCG where they were centrifuged, and stored at -80°C until analysis.

Cortisol samples were analyzed by means of online-solid phase extraction in combination with isotope dilution liquid chromatography-tandem mass spectrometry (LC-MS/MS), which has a broad analyte compatibility and high analytical performance. Details can be obtained from the corresponding author upon request. In short, 250uL of saliva was used for the analysis and deuterated cortisol was used as internal standard. All samples of one participant were assayed in the same batch. Mean intra- and inter-assay coefficients of variation were below 10%. The quantification limit for cortisol was 0.1 nmol/L.

Alpha-amylase was analysed at the same day at the general haematology and chemistry Lab, UMCG, by means of an enzymatic colorimetric analysis, according to the International Federation of Clinical Chemistry (IFCC) method, Roche Modular P, Mannheim, Germany. All samples of one participant were assayed in the same batch. The mean intra- and inter-assay coefficients of variation were 0.9% and 1.0%, respectively.

### Lifestyle variables

A number of items were included to assess variables suspected to influence cortisol or α-amylase levels, i.e. items on food intake, substance use, and exercise. Participants indicated whether, in the previous 1.5h, they ate/drank: 1) bananas / chocolate / dairy products; 2) a meal; 3) a sugary or fatty snack; 4) an alcoholic drink; 5) a caffeinated drink; 6) nothing of the before-mentioned options but something else; 7) nothing of the before-mentioned options and nothing else. They were allowed to enter 3 of these categories. Categories 1, 2, and 3 were merged into one category, because these all include nutritional products considered to have stimulating effects on cortisol secretion and presumably α-amylase secretion [10,27]. Participants also indicated whether they used any of the following substances in the past six hours: 1) alcohol; 2) caffeine; 3) nicotine; 4) stimulating drugs (e.g. cocaine); 5) calming drugs (e.g. sleeping pills); 6) cannabis. Finally, they indicated whether they engaged in exercise during the past six hours. Exercise was defined as any physical activity done in leisure time with being active as the main purpose. Actigraphy data from the ActiCal was used to calculate wake-up time. Using an algorithm developed by Sadeh and colleagues, the probability of sleep was calculated for every 1-minute epoch [28]. Counting backwards from the moment when participants filled out their morning questionnaire (as registered by the PsyMate), the first minute of being awake after at least 6 minutes of sleep was determined as the moment of awakening. If participants filled out the morning questionnaire within 60 minutes after waking up, the variable ‘recent awakening’ was given a score ‘1’. Otherwise, the variable was given a score ‘0’ [15]. All other lifestyle items were dummy-coded as well, resulting in a total of 12 dichotomous covariates.

### Person characteristics

Age, gender, completed education (0 = primary education, 1 = lower secondary education, 2 = upper secondary education/vocational education, 3 = university/college education), BMI, and smoking status were based on self-report. DSM diagnosis and information on previous depressive episodes were obtained from the CIDI interview. Average bedtime on work and free days was assessed with the MCTQ. Medication use was assessed weekly throughout the study.

### Statistical analysis

On average, participants had 4.1 (4.6%) missing or disqualified cortisol and α-amylase samples, respectively. Saliva samples were disqualified if they were collected outside the one-hour time window during which the electronic diary could be filled out. In a few cases, participants sampled saliva after this one-hour time window using a paper diary, but clearly stated the time of sampling. If this sampling time was < 30 minutes after the time window, (i.e. <1.5 hour after the beep), samples were also considered valid, because we preferred real values over imputed values. On average, participants had 6.8 (7.5%) missing values for the diary lifestyle variables. Actigraphy data was used to construct the variable ‘recent awakening’. Three participants had missing actigraphy data, because of technical problems at the last 9 or 10 days of their study. This resulted in a total of 28 missing values for ‘recent awakening’ (0.1% for the total sample). Missing data were imputed by means of Expectation-Maximization imputation, based on all variables in the dataset that were significantly associated with the variables containing missing values, as well as lagged values of cortisol and α-amylase (6 lags) [29]. In all analyses, we discarded the first 20 days of participant D12 because of extreme cortisol values, presumably due to an infection. Values returned to a more normal range after day 20, around when D12 had started taking antibiotics. This left 30 measurements valid measurements, which is the minimal requirement for performing time-series analysis, which will be explained next [30].

For every individual, the cortisol and α-amylase time series were corrected for the influence of the lifestyle variables by means of time-series regression analysis [31]. This technique is specifically suited for the assessment of within-person associations for each individual separately, based on a large number of measurements over time. With this technique, the influence of the lifestyle variables can be assessed while adjusting for autocorrelation, which is often present in repeated observations from the same individual. Temporal variation in the measures under study is required. Therefore, variables that occurred less than 5 times during the 30-day study period or were always present were excluded from the analysis. We included dummy variables for morning and afternoon to account for structural daytime differences in levels. If necessary, the measurement number was included to render a series stationary. Any residual autocorrelation was identified using (partial) autocorrelation functions (ACFs and PACFs) and Ljung-Box tests, and removed by fitting autoregressive moving average (ARMA) models to the residuals (see S1 Text, for an example of an ARMA model, and the accompanied ACF, PACF and Ljung-Box test. Models were implemented using the SPSS 20 Forecasting module. Adjusted cortisol and α-amylase values were computed by subtracting model estimates of the covariates from the unadjusted values.

Differences between the depressed and the non-depressed group were assessed by multilevel analyses with the crude and corrected cortisol and α-amylase levels, daily slope of cortisol, and ratio of α-amylase over cortisol as dependent variables. Daily slope represents the averaging of the difference between morning and afternoon levels and the difference between afternoon and evening levels per day. The ratio of α-amylase over cortisol was constructed by dividing α-amylase levels by cortisol levels at the same time point. Depression status was included in the model as a fixed factor. To account for daily cycles and time trends, variables for day segment and time were included as both fixed and random factors. Random intercepts and slopes were included to account for between-subjects variability. Models with different covariance structures were fitted using restricted maximum likelihood estimation and the most optimal model was chosen based on the Aikaike Information Criterion (AIC). The best model was finally estimated with maximum likelihood estimation. Because cortisol and α-amylase distributions were skewed, bootstrapped confidence intervals were computed, using 1000 bootstrap samples. Bootstrapping has the advantage that is does not assume any specific distribution of the data. In a second step, to get an impression of the influence of antidepressant use on the results, the use of psychotropic drugs or Saint John’s wort was added as a fixed factor to the multilevel models.

Subsequently, we compared matched pairs of depressed and non-depressed individuals with regard to their cortisol and α-amylase measures. Thereafter, we calculated the (binomial) probability that the percentage of comparisons in which depressed individuals had increased cortisol and α-amylase levels, a flatter slope and a larger α-amylase over cortisol ratio, compared to their non-depressed match, significantly differs from chance (50%). Furthermore, crude and adjusted cortisol and α-amylase time series were visually inspected and examined using descriptive statistics (means and variances). For each individual series, the presence of time trends over the month and differences between day segments was assessed with time-series regression analysis, using dummy variables for day segment (morning and afternoon compared to evening) and a time variable as predictors. Again, any autocorrelation was removed by fitting ARMA models to the residuals.

## Results

### Group characteristics

Demographic, clinical and biological characteristics of the 15 depressed and 15 non-depressed participants are described in Table 1. As expected, significant differences between groups were found for pre-BDI scores (t = -10.73, p<0.001) and post-BDI scores (t = -5.02, p<0.001). No other significant group differences were found. Of the depressed group, 11 were currently depressed, and 4 were in remission since 2–4 weeks. Furthermore, 7 depressed participants were on psychotropic medication throughout the study period, and 2 used Saint John’s wort (see S1 Table, for a complete overview of medication and therapy use). Average cortisol and α-amylase levels ranged from 1.78 to 7.22 nmol/l and from 11.7 to 656.7 U/ml, respectively.

**Table 1:** Characteristics of depressed and non-depressed groups.

Characteristics	Depressed	Non-depressed
	Mean (SD)	Mean (SD)
**Demographic**		
Gender (% female)	73.3	73.3
Age	35.9 (10.5)	35.1 (8.4)
BMI	23.6 (4.5)	22.5 (2.8)
Smoking status (% smoking)	20.0	20.0
Highest completed education (0–4)	2.4 (0.5)	2.5 (0.5)
**Daily schedule**		
Average bedtime work days	22:44 (0:56)	23:00 (1:10)
Average bedtime free days	23:25 (1:19)	23:53 (1:07)
Evening beep time PsyMate	22:06 (1:03)	22:15 (0:50)
**Clinical**		
History (% with prior episodes)	66.7	6.7
BDI pre	30.8 (9.8)	2.4 (3.1)
BDI post	23.1 (14.4)	3.6 (4.3)
**Biological**		
Average cortisol levels during study	3.92 (1.29)	3.50 (1.39)
Average α-amylase levels during study	224.1 (181.1)	173.9 (120.0)

### Group comparisons

Cortisol and α-amylase levels were significantly higher and the ratio of α-amylase over cortisol was significantly larger in the depressed than in the non-depressed group (Table 2). There was a trend towards a steeper daily cortisol slope in the depressed group (p = .075). Afternoon and evening cortisol levels were significantly lower than morning levels. In contrast, α-amylase levels and the ratio of α-amylase over cortisol were significantly higher in the afternoon and evening and increased over the month, while cortisol levels did not change significantly.

**Table 2:** Results of multilevel analysis of relationship between depression status and cortisol and α-amylase measures.

	Bootstrapped estimates (95% CI)			
*Fixed effects*	Cortisol (nmol/l)	Alpha-amylase (U/ml)	Daily slope cortisol	Ratio α-amylase over cortisol
Intercept	6.55 (6.20–6.90) **	125.2 (108.6–141.9) **	-2.67 (-2.91 –-2.43) **	24.0 (15.0–33.0) **
Depression	0.30 (0.04–0.56)*	54.8 (33.2–76.4) **	-0.31 (-0.65–0.03)^†^	12.2 (3.1–21.3) **
Time	-0.00 (-0.00–0.00)	0.3 (0.1–0.5) **	0.00 (-0.00–0.01) ^†^	0.2 (0.0–0.3) *
Afternoon	-3.61 (-3.89 –-3.32)**	55.2 (43.7–66.7) **	-	58.7 (52.1–65.4) **
Evening	-5.27 (-5.55 –-5.00) **	38.9 (27.3–50.4) **	-	173.1 (161.8–184.5) **

Note: CI = confidence interval.

* p<0.05

**p<0.01

^†^p<0.10

After controlling individual time series for lifestyle factors, the relationship between cortisol levels and depression status was no longer significant, neither was the relationship of depression to the ratio of α-amylase over cortisol (for an overview of the individual-specific influences of lifestyle factors on cortisol and α-amylase, see S2 Table and S3 Table, respectively). The association between depression status and α-amylase levels remained significant. Furthermore, the relationship with the daily slope became significant (see S4 Table, for the results of the mixed-model analysis adjusted for lifestyle factors).

Use of antidepressant medication (psychotropic drugs or Saint John’s wort) was positively associated with both cortisol (B = 0.86, p < .001) and α-amylase (B = 115.8, p < .001), and resulted in a reduced and non-significant relationship of depression with cortisol (B = -0.09, p = .47) and α-amylase (B = -4.99, p = .76). Antidepressant medication had no influence on the daily cortisol slope (B = 0.21, p = .45), or the α-amylase over cortisol ratio (B = -1.95, p = .70).

### Matched comparisons

Depressed individuals had higher cortisol levels in more than half of the comparisons (60%), but had higher α-amylase levels, a steeper daily slope and a larger ratio of α-amylase over cortisol in *less than* half of the comparisons (47%, 40%, 40%; see S5 Table, for a listing of all matched comparisons). Adjustment for lifestyle factors led to slightly different percentages for these comparisons, ranging between 40% and 53% (see S6 Table, for a listing of all matched comparisons). None of the percentages differed significantly from chance (50%).

### Characteristics of individual cortisol and α-amylase time series

#### Cortisol

**Fig 1:**
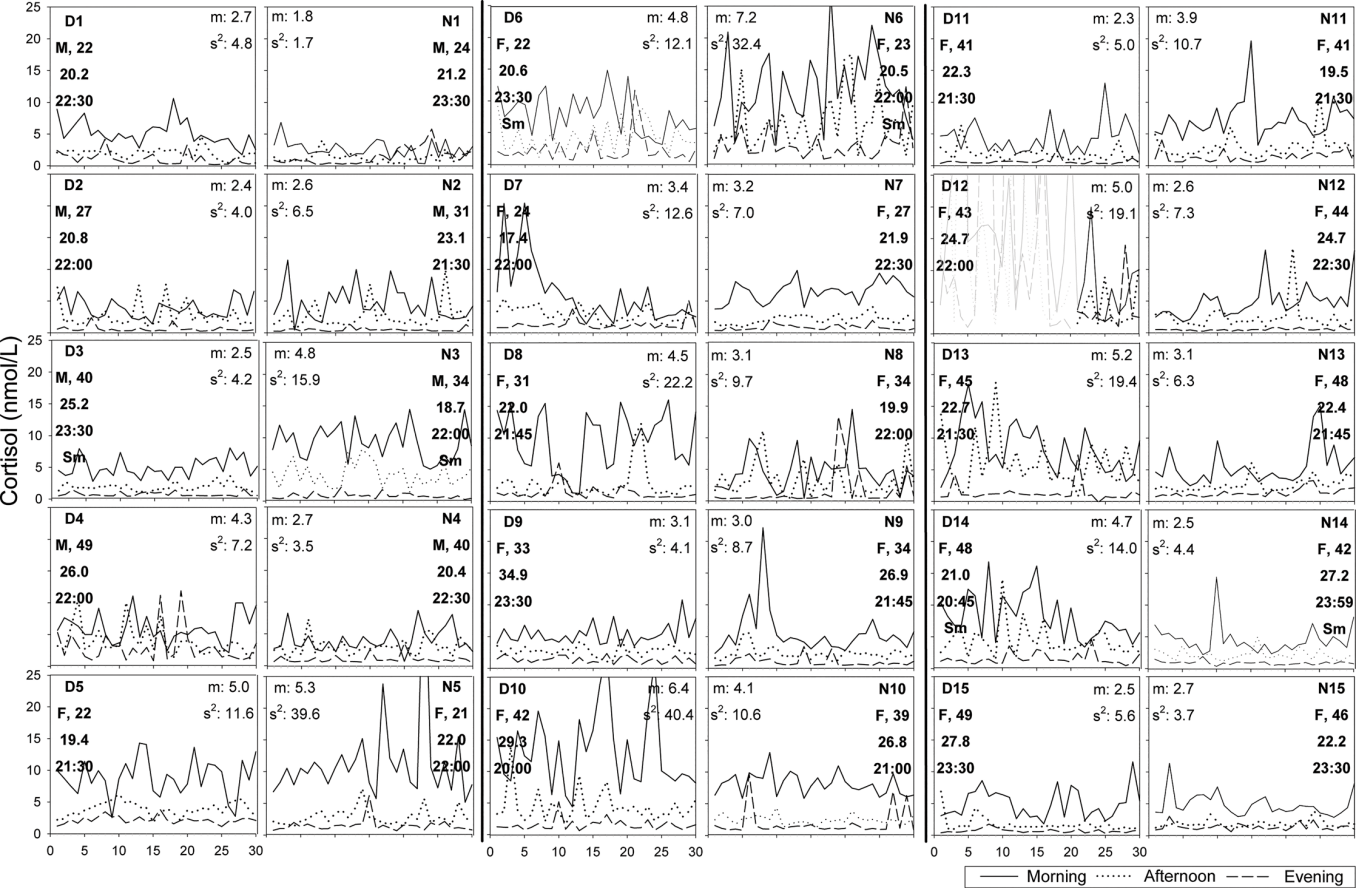
Cortisol patterns over 30 days stratified by beep-time (morning, afternoon and evening values). Graphs of pair-matched individuals are placed alongside each other (e.g. D1 and N1). In the corner of the graphs the following information is displayed: identification number (D = depressed, N = non-depressed), gender (F = female, M = male), age in years, BMI, evening beep-time, and, if smoking ‘Sm’. Descriptive statistics at the opposite side of the graphs display m, the mean of the series and s^2^, the variance of the series.

The individual graphs revealed substantial interindividual differences in levels and variances of cortisol, both within and between the depressed and non-depressed group (Fig 1). Mean levels ranged from 1.8 to 7.2 nmol/l, and variances from 1.7 to 40.4. While in most individuals cortisol fluctuated around a relatively constant mean level, in some individuals the levels significantly increased (N7, N12, N13) or decreased (D1, D7, D13, D14) over the month.

Cortisol levels were generally highest in the morning and lowest in the evening. However, for some individuals, the differences between afternoon and evening levels were less pronounced and non-significant (D4, N5, D7, D8, N10, N15). For one individual (N1), afternoon levels were on average lower than evening levels.

#### Alpha-amylase

**Fig 2:**
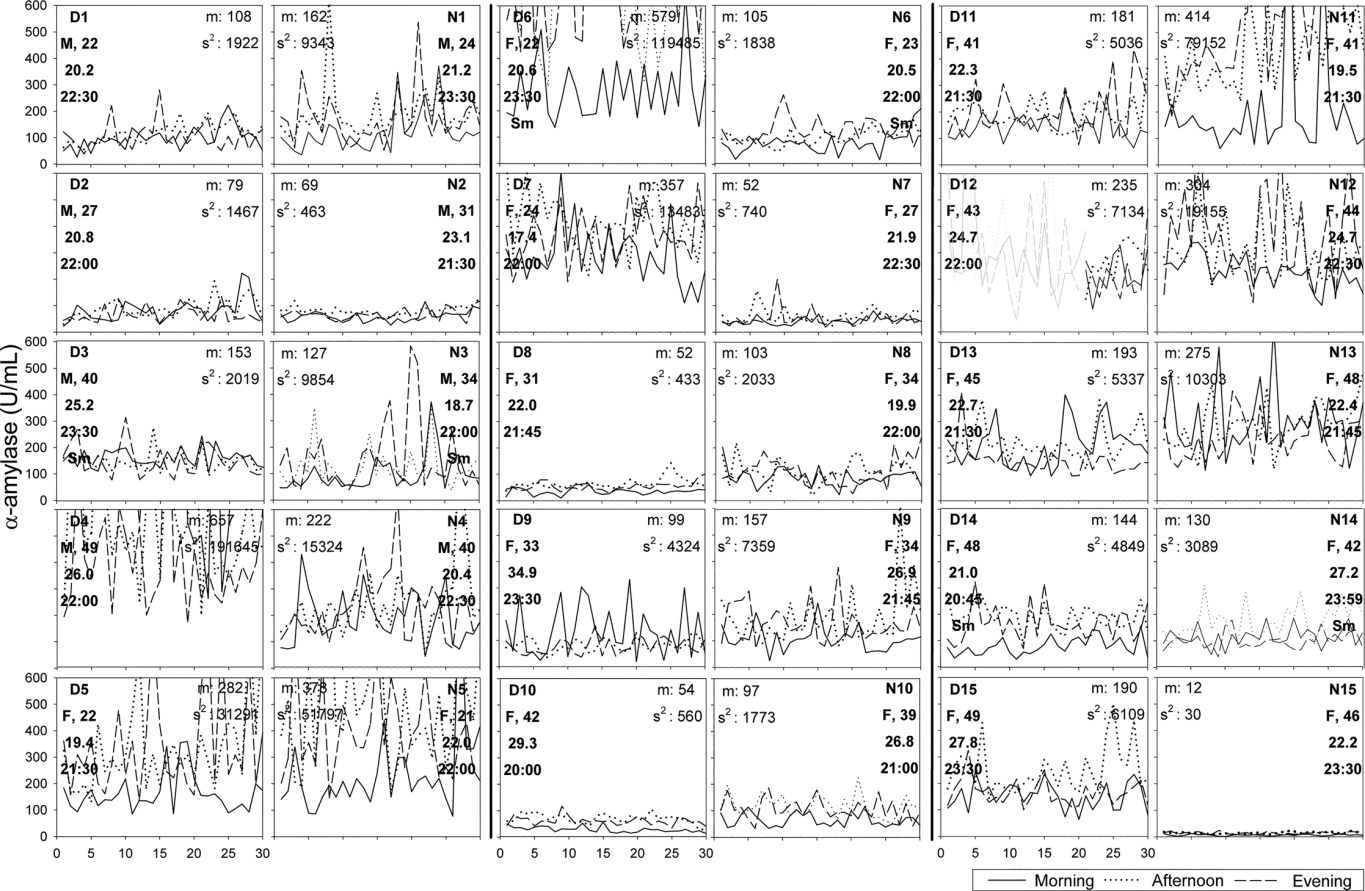
Alpha-amylase patterns over 30 days stratified by beep-time (morning, afternoon and evening values). Graphs of pair-matched individuals are placed alongside each other (e.g. D1 and N1). In the corner of the graphs the following information is displayed: identification number (D = depressed, N = non-depressed), gender (F = female, M = male), age in years, BMI, evening beep-time, and, if smoking ‘Sm’. Descriptive statistics at the opposite side of the graphs display m, the mean of the series and s2, the variance of the series.

Also the individual graphs of α-amylase revealed substantial interindividual differences in levels and variances, both within and between the groups (Fig 2). Mean levels of α-amylase ranged from 12 to 657 U/ml, and variances from 30 to 191645. Time trends in α-amylase were found in several individuals (increase: D1, D2, D5, D8, N6, N11, N14; decrease: D10, N10).

In most individuals, morning α-amylase levels were lowest, and afternoon levels were highest. However, in some individuals highest levels were found in the evening (D5, D6, D11, N3, N6, N8, N11, N12); in others (D3, D4, D9, N13) in the morning.

## Discussion

This is the first study to address the relationship between depression and cortisol and α-amylase secretion patterns in daily life by comparing group- and individual-level results. Our analyses suggest that cortisol and α-amylase secretion is related to depression at the group level: on average, the depressed group showed somewhat higher cortisol, higher α-amylase levels, a steeper diurnal rhythm of cortisol, and a larger ratio of α-amylase over cortisol than the non-depressed group. However, generalization of these findings to individuals is troublesome, because a comparison of matched depressed and non-depressed individuals indicated that the differences within pairs were not consistent and frequently opposite to the group-level results. Further, visual inspection of the individual time series revealed substantial intra- and interindividual variation in cortisol and α-amylase secretion patterns, regardless of depression status, making it difficult to discriminate depressed from non-depressed individuals on the basis of their cortisol or α-amylase levels.

After individual-specific correction for lifestyle factors, the differences between the depressed and non-depressed group remained significant with regard to α-amylase levels, and became significant—previously there was a trend—with regard to the daily slope of cortisol. In contrast, the association of depression to cortisol and the ratio of α-amylase over cortisol disappeared. Hence, daily-life fluctuations in cortisol and α-amylase, in particular due to lifestyle, are a potential source of inconsistencies in the present literature. We further found that antidepressant medication influenced cortisol and α-amylase positively, and largely negated the relationship with depression. If anything, we expected a negative effect of antidepressant medication on the ANS [32,33] and the HPA axis [34,35], although some studies also show mixed results [36], but we could not explore this association further because of sample size limitations.

The significant differences found at the group-level were not consistently replicated in the matched comparisons of depressed and non-depressed individuals. In fact, with regard to α-amylase, the daily slope of cortisol, and the ratio of α-amylase over cortisol, the majority of comparisons were in the opposite direction of the group results. These discrepancies between the group- and individual-level results may be due to the presence of individuals with extreme values; two individuals (D4 and D6) had very high α-amylase levels compared to the other individuals (see Fig 2). These individuals showed relatively high values throughout the month, meaning that these values were not simply outliers or due to measurement error. The high levels of these individuals may explain why *on average* the depressed group had higher α-amylase levels than the non-depressed group, while the *majority* of depressed individuals had *lower* α-amylase levels than their non-depressed match partner.

Extreme values in the sample are not the only reason for the divergence between the findings at the group and the individual level. Visual inspection of the individual time series of cortisol and α-amylase showed that the interindividual variation in levels and variances was remarkably large, especially in the light of the estimated group differences. Also the course over the month and daily rhythms were not consistent across individuals. This was especially true for α-amylase, where some individuals showed quite opposite daily rhythms from what is known to be typical (i.e. low morning values, which increase until the afternoon and stabilize thereafter [37]. Thus, even with a sizeable number of measurements, all statistical parameters of the time series of cortisol and α-amylase were highly heterogeneous across individuals, both within and across groups, regardless of correction for lifestyle factors (see appendix S1 Fig and S2 Fig, for the lifestyle-adjusted time-series).

Most psychophysiological research into depression is group-based, and is performed under the assumption that what is found on average is also true in general, or at least true for the majority of individuals. The debate about the use of nomothetic research designs to find generalities in individual functioning has been going on for some time [18,38,39]. Lamiell (1981) has argued that nomothetic studies show what holds among the participants on average, but do not reveal what is common to all of those participants. The reason is that obtained results certainly do not hold for at least some of the subjects and quite possibly do not hold for most of them. The finding that cortisol levels are not increased in all depressed individuals is not new, and has been acknowledged many times before (e.g. [40,41]). But beyond that, our results exemplify that group results may not even hold for the majority of individuals. This calls for caution when generalizing group results to the individual level. Lamiell further argued that, since in psychology the objects of study are individual organisms, persons should be the entity of study, not aggregates of persons. This requires idiographic studies with multiple repeated measurements that are replicated in many individuals to find commonalities among them. A similar line of reasoning has been adopted by Molenaar and Campbell [42], who argued that group results regarding processes that fluctuate over time can hardly ever be generalized to the individual level, because this requires that statistical characteristics are invariant across subjects (homogeneity) and time (stationarity). If these so-called ergodicity assumptions are not met, the jump from the population to the individual level cannot be validly made.

Our study had a number of limitations. First of all, we collected saliva using Salivettes, which is, compared to the passive drooling technique, a theoretically suboptimal technique for assaying α-amylase. However, because the latter technique requires much practice and close monitoring, in ecological momentary assessment studies the Salivette is still the preferred option [25]. Furthermore, the highest inter-correlation between cortisol and α-amylase is found when measured 10–20 minutes apart, while we assessed cortisol and α-amylase simultaneously [43]. This may have resulted in an underestimation of the relationship between depression and the α-amylase/cortisol ratio. Also, because the assessment of lifestyle behaviors and saliva sampling happened simultaneously, the true direction of the relationship cannot be inferred. Therefore, both corrected and uncorrected results were considered to be informative, and hence, presented. Relating to this, we lacked information on quantities and exact timing of the factors, which, if available, might have improved the estimation of the effects of lifestyle factors. Another point is that several depressed individuals used antidepressants (see S1 Table, for a complete overview of medication and therapy use), which seemed to affect functioning of the stress system at the group-level, and may do so within individuals as well. Please note, however, that controlling for antidepressant medication reduced the association of depression with cortisol and α-amylase, not increased it, so a better control of medication use would not have changed our main conclusion that cortisol and α-amylase secretion patterns are highly subject-specific. Furthermore, because of the small sample size, it is difficult to disentangle effects of antidepressant use and remission; the latter, operationalized with the post-study BDI score, also reduced the association between depression and cortisol and α-amylase (see S7 Table). In addition, despite a strict matching protocol, some within-pair differences in BMI and age remained. A sensitivity analysis indicated that controlling for BMI or age did not influence the relationship between depression and cortisol and α-amylase measures at the group level (see S8 Table and S9 Table). However, it still might have influenced the results of the pair-matched comparisons. Other factors that we did not match on may have influenced the pair-matched comparisons as well. However, for practical reasons we confined our matching protocol to gender, age, BMI and smoking behavior. Further, there was a small but significant trend over the month at the group level. This is because for seven of the thirty individuals, there was a significant positive time trend. We cannot discern whether these subjects became less strict with the sampling regulations or that they experienced more stressful events. Finally, we did not control for ultradian rhythmicity, which may have increased intra- and interindividual variability. However, real-time assessment of cortisol, which is necessary to assess ultradian rhythmicity, is currently only feasible for a maximum of 24 hours [44]. Hence, the longer interval between the measurements was a trade-off with the long study period of 30 days.

### Conclusions

To conclude, we combined a nomothetic and idiographic approach to study the relationship between cortisol, α-amylase, and depression in daily life. Our findings at the group level were largely in line with what is considered ‘consensus’ in the literature. However, intra- and interindividual variability in cortisol and α-amylase was surprisingly large, and the group results did not correspond well to the results at the individual level. Findings of studies on this topic should therefore be interpreted with care, because in clinical practice the focus is on individuals instead of groups. A bottom-up approach, in which relationships are studied within individuals over time and thereafter compared between individuals to find meaningful subgroups, may be a more promising method to detect (individual) stress-related mechanisms in depression.

## Supporting Information

S1 Text ARMA modelling. (DOCX)

S1 Table Antidepressant medication/therapy use throughout the study period. (DOCX)

S2 Table Effects of lifestyle variables on cortisol. (DOCX)

S3 Table Effects of lifestyle variables on α-amylase. (DOCX)

S4 Table Results of multilevel analysis of the relationship between depression status and adjusted cortisol and α-amylase measures. Note: CI = confidence interval. * p<0.05; **p<0.01; ^†^p<0.10.
(DOCX)

S5 Table Matched-pair comparisons of crude cortisol and α-amylase measures. Note: D = depressed match, N = non-depressed match, > = bigger than, < = smaller than.
(DOCX)

S6 Table Matched-pair comparisons of adjusted cortisol and α-amylase measures. D = depressed match, N = non-depressed match, c = controlled for lifestyle factors, > = bigger than, < = smaller than.
(DOCX)

S7 Table Results of multilevel analysis of the relationship between depression status and crude cortisol and α-amylase, corrected for post-BDI score. Note: CI = confidence interval. BDI = Beck Depression Inventory. * p<0.05; **p<0.01; ^†^p<0.10
(DOCX)

S8 Table Results of multilevel analysis of the relationship between depression status and crude cortisol and α-amylase measures, corrected for BMI. Note: CI = confidence interval. BMI = Body Mass Index. * p<0.05; **p<0.01; ^†^p<0.10
(DOCX)

S9 Table Results of multilevel analysis of the relationship between depression status and crude cortisol and α-amylase measures, corrected for age. Note: CI = confidence interval. BMI = Body Mass Index. * p<0.05; **p<0.01; ^†^p<0.10
(DOCX)

S1 Fig Adjusted cortisol patterns over 30 days stratified by beep-time (morning, afternoon and evening values). Graphs of pair-matched individuals are placed alongside each other (e.g. D1 and N1). In the corner of the graphs the following information is displayed: identification number (D = depressed, N = non-depressed), gender (F = female, M = male), age in years, BMI, evening beep-time, and, if smoking ‘Sm’. Descriptive statistics at the opposite side of the graphs display m, the mean of the series and s^2^, the variance of the series.
(EPS)

S2 Fig Adjusted α-amylase patterns over 30 days stratified by beep-time (morning, afternoon and evening values). Graphs of pair-matched individuals are placed alongside each other (e.g. D1 and N1). In the corner of the graphs the following information is displayed: identification number (D = depressed, N = non-depressed), gender (F = female, M = male), age in years, BMI, evening beep-time, and, if smoking ‘Sm’. Descriptive statistics at the opposite side of the graphs display m, the mean of the series and s^2^, the variance of the series.
(EPS)

S1 Dataset (XLSX)
