# Supplementary material for: Cortisol and α-Amylase Secretion Patterns between and within Depressed and Non-Depressed Individuals
Source: PLoS One. 2015 Jul 6;10(7):e0131002. doi: 10.1371/journal.pone.0131002 (PMC4492984; doi:10.1371/journal.pone.0131002)

**S1 Text.** **ARMA modelling.**

As an example, the initial ARMA model (Table A) and the final ARMA model (Table B) for participant N13 are presented below. The initial model contains dummy variables for morning and afternoon, as well as measurement number. As mentioned in the Methods section, dummy variables for time of day are always added because cortisol and a-amylase show daily rhythms. This is also true for this participant: cortisol is significantly higher in the morning and afternoon, compared to the evening (reference category). In addition, measurement number is included to assess whether there is a time trend in the data. From the p-value it can be taken that this is indeed so. Hence, to render the series stationary, measurement number is maintained in the model. According to the Ljung-Box test, there is residual autocorrelation (p<0.05). This can also be seen from the autocorrelation function (ACF) (For more information regarding the difference between the ACF and PACF, please see Chatfield, Chris. *The analysis of time series: an introduction*. CRC press, 2013: Chapter 4). Specifically, at lag 3 there is autocorrelation present, where the bar crosses the 95% confidence interval. Therefore, the next step is to include an autoregressive (AR)3 term to the model.

Table A. Initial ARMA model

| **Model Statistics** | | | | | | |
| --- | --- | --- | --- | --- | --- | --- |
| Model | Number of Predictors | Model Fit statistics | Ljung-Box Q(18) | | | Number of Outliers |
|  |  | Stationary R-squared | Statistics | DF | Sig. |  |
| cortisolnmolL-Model_1 | 3 | ,525 | 31,588 | 18 | ,025 | 0 |

| **ARIMA Model Parameters** | | | | | | | | |
| --- | --- | --- | --- | --- | --- | --- | --- | --- |
|  | | | | | Estimate | SE | T | Sig. |
| cortisolnmolL-Model_1 | cortisolnmolL | No Transformation | Constant | | ,288 | ,459 | ,628 | ,531 |
|  | Measurement number | No Transformation | Numerator | Lag 0 | ,024 | ,007 | 3,285 | ,001 |
|  | Dummy morning | No Transformation | Numerator | Lag 0 | 4,132 | ,456 | 9,058 | ,000 |
|  | Dummy afternoon | No Transformation | Numerator | Lag 0 | 1,278 | ,456 | 2,803 | ,006 |


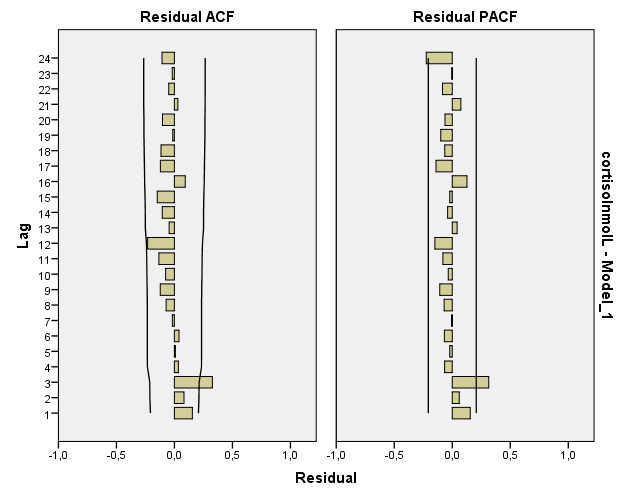


Adding an AR3 term was sufficient for removing residual autocorrelation. This individual step is not shown. Instead, the final model is shown, where the lifestyle variables are also added. In the final model, it can be seen that the influence of AR3 on cortisol is significant. Furthermore, the Ljung-Box test is no longer significant, indicating that adding an AR3 term was successful to remove autocorrelation. This is also suggested by the ACF: there is no significant autocorrelation at any lag. Five lifestyle variables were added to the model. These variables occurred >5 times during the study period. The influence of exercise is significant at the p<0.10 level. No other variables have a significant influence on cortisol.

Table B. Final ARMA model.

| **Model Statistics** | | | | | | |
| --- | --- | --- | --- | --- | --- | --- |
| Model | Number of Predictors | Model Fit statistics | Ljung-Box Q(18) | | | Number of Outliers |
|  |  | Stationary R-squared | Statistics | DF | Sig. |  |
| cortisolnmolL-Model_1 | 8 | ,620 | 14,246 | 17 | ,650 | 0 |

| **ARIMA Model Parameters** | | | | | | | | |
| --- | --- | --- | --- | --- | --- | --- | --- | --- |
|  | | | | | Estimate | SE | T | Sig. |
| cortisolnmolL-Model_1 | cortisolnmolL | No Transformation | Constant | | 1,015 | ,759 | 1,338 | ,185 |
|  |  |  | AR | Lag 3 | ,282 | ,110 | 2,560 | ,012 |
|  | Measurement number | No Transformation | Numerator | Lag 0 | ,026 | ,009 | 2,751 | ,007 |
|  | Dummy morning | No Transformation | Numerator | Lag 0 | 3,618 | ,629 | 5,755 | ,000 |
|  | Dummy afternoon | No Transformation | Numerator | Lag 0 | ,870 | ,638 | 1,365 | ,176 |
|  | Caffeine use recent | No Transformation | Numerator | Lag 0 | -,130 | ,565 | -,230 | ,819 |
|  | Cafeïne use recent | No Transformation | Numerator | Lag 0 | -,652 | ,479 | -1,362 | ,177 |
|  | Caloric-rich food recent | No Transformation | Numerator | Lag 0 | ,508 | ,417 | 1,220 | ,226 |
|  | Other food recent | No Transformation | Numerator | Lag 0 | -,455 | ,435 | -1,045 | ,299 |
|  | Exercise | No Transformation | Numerator | Lag 0 | -,773 | ,431 | -1,791 | ,077 |


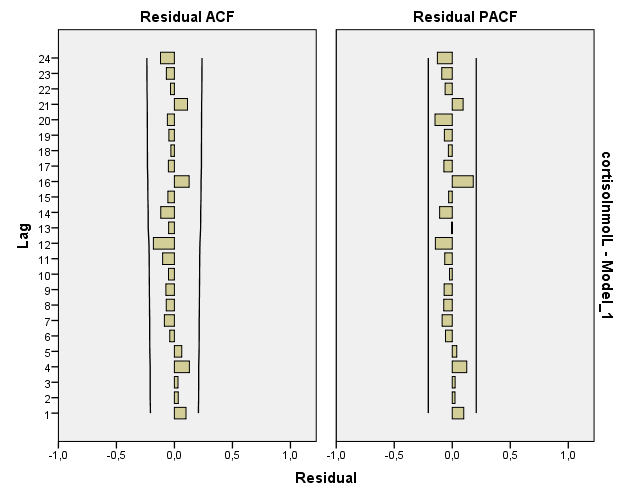

Supplement: S1 Text — (DOCX) [file pone.0131002.s001.docx]
