# Supplementary material for: Cortisol and α-Amylase Secretion Patterns between and within Depressed and Non-Depressed Individuals
Source: PLoS One. 2015 Jul 6;10(7):e0131002. doi: 10.1371/journal.pone.0131002 (PMC4492984; doi:10.1371/journal.pone.0131002)
Supplement: S1 Table — (DOCX) [file pone.0131002.s002.docx]

**S1 Table. Antidepressant medication/therapy use throughout the study period**

| **ID** | **Antidepressant medication** | **Period** |
| --- | --- | --- |
| D1 | Vitamin D3 50.000 IU | Every week |
| D2 | Light therapy | Every Monday to Friday |
| D3 | Oxazepam 30 mg | Day 20 – 21 |
| D4 | Imipramine 25 mg  Lithium 400 mg | Day 1 – 30  Day 1 – 30 |
| D5 | Escitalopram 20 mg | Day 1 – 30 |
| D6 | Diazepam 5 mg | Day 12 – 13 |
| D7 | Sertraline 25-75 mg | Day 1 – 30 (increasing dosage) |
| D8 | Amitriptyline 25 mg | Day 23 – 30 |
| D9 | Quetiapine 300 mg  Multivitamins  Vitamin D 600 mg | Day 1 – 30  Day 1 – 30 (every other day)  Day 1 – 30 (every other day) |
| D10 | Mirtazapine 30 mg | Day 1 – 30 |
| D12 | Quetiapine 200 mg  Venlafaxine 300 mg | Day 1 – 30  Day 1 – 30 |
| D13 | Sertraline 50 mg  Sertraline 150 mg  Quetiapine 25 mg | Day 1 – 28  Day 29 – 30  Day 1 – 30 |
| D14 | St. John’s wort 900 mg  Oxazepam 50 mg | Day 1 – 30  Day 1 – 30 |
| D15 | St. John’s wort 900 mg | Day 1 – 30 |
| N13 | Citalopram 20 mg | Day 1 – 11 |
