# Supplementary material for: Cortisol and α-Amylase Secretion Patterns between and within Depressed and Non-Depressed Individuals
Source: PLoS One. 2015 Jul 6;10(7):e0131002. doi: 10.1371/journal.pone.0131002 (PMC4492984; doi:10.1371/journal.pone.0131002)
Supplement: S3 Table — (DOCX) [file pone.0131002.s004.docx]

**S3 Table. Effects of lifestyle variables on α-amylase.**

| ID | Caffeine | Caffeine recent | Alcohol | Alcohol recent | Nicotine | Caloric-rich food | Other food | Exercise | Recent awakening |
| --- | --- | --- | --- | --- | --- | --- | --- | --- | --- |
| *Depressed* | | | | | | | | | |
| 1 | + | - | + | - |  | - | + | + |  |
| 2 | + | + |  |  |  | - |  |  |  |
| 3 | * | + | - | + | * | - | - | + |  |
| 4 | + | + | + | - |  | - | + |  |  |
| 5 |  |  |  |  |  | - | + | - | + |
| 6 | + | - | * | - | + | + | - |  | - |
| 7 |  |  |  |  |  | + |  |  | - |
| 8 |  |  | - | + |  | - | - | - | - |
| 9 | + | - |  |  |  | + |  | - | + |
| 10 | - |  | + |  |  | + | - | + | - |
| 11 | - |  |  |  |  | + | + | - |  |
| 12 |  |  |  |  |  |  |  |  | - |
| 13 | - | - |  |  |  | + | + | + | - |
| 14 |  |  | + | - | + | + | + | + | + |
| 15 |  |  |  |  |  | - | - |  | - |
| *Non-depressed* | | | | | | | | | |
| 1 | + | - |  |  |  |  |  |  | - |
| 2 |  |  |  |  |  |  | - |  | - |
| 3 | + | - | + | + | - | - | - |  | + |
| 4 | + |  |  |  |  | + |  |  | + |
| 5 | + |  |  |  |  | - | - |  |  |
| 6 | - | + |  |  | + | - | - |  | + |
| 7 | - | + | - | - |  | - | + | - |  |
| 8 | + |  |  |  |  | - |  |  |  |
| 9 | + | - | + | - |  | + | + | + |  |
| 10 | + |  |  |  |  | + | + |  | - |
| 11 |  |  |  |  |  | + | + | - | - |
| 12 | + | - | - | + |  | + | + |  | - |
| 13 | - | - |  |  |  | - | - | + |  |
| 14 |  | - |  |  | + | - | + |  |  |
| 15 | + |  | * | - |  |  | + | - | - |

Note: + = Positive influence, - = Negative influence, White cells = Corresponding variable is not added to the ARMA model, because it was absent or present <5 times, Light green cells = Non-significant positive influence, Middle green cells = Positive influence significant at the p<0.05 – 0.10 level, Dark green cells = Positive influence significant at the p<0.05 level, Light red/pink cells = Non-significant negative influence, Middle red/pink cells = Negative influence significant at the p<0.05 – 0.10 level, Dark red/pink cells = Negative influence at the p<0.05 level, * = Corresponding variable is not added to the ARMA model, because it was always present (invariant) or co-varying with another variable.
