# Supplementary material for: Cortisol and α-Amylase Secretion Patterns between and within Depressed and Non-Depressed Individuals
Source: PLoS One. 2015 Jul 6;10(7):e0131002. doi: 10.1371/journal.pone.0131002 (PMC4492984; doi:10.1371/journal.pone.0131002)
Supplement: S4 Table — Note: CI = confidence interval. * p<0.05; **p<0.01; †p<0.10. (DOCX) [file pone.0131002.s005.docx]

**S4 Table. Results of multilevel analysis of the relationship between depression status and adjusted cortisol and α-amylase measures**

| **Variables** | **Bootstrapped estimates (95% CI)** | | | |
| --- | --- | --- | --- | --- |
| *Fixed effects* | **Cortisol (nmol/l)** | **Alpha-amylase (U/ml)** | **Slope cortisol** | **Ratio α-amylase over cortisol** |
| Intercept | 7.10  (6.12 – 8.08)** | 123.6  (111.0 – 138.5)** | -2.65  (-2.88 – -2.42)** | 17.3  (-110.0 – 144.6) |
| Depression | -0.13  (-0.78 – 0.51) | 44.8  (28.0 – 61.6)** | -0.57  (-0.88 – -0.27)** | 62.2  (-120.7 – 245.1) |
| Time | -0.00  (-0.01 – 0.00) | 0.3  (0.1 – 0.5)** | 0.00  (-0.00 – 0.01) | -0.2  (-4.0 – 3.5) |
| Beep afternoon | -3.87  (-4.39 – -3.35)** | 55.5  (44.5 – 66.6)** | – | 37.8  (-0.9 – 76.5) ^†^ |
| Beep evening | -5.62  (-6.58 – -4.65)** | 43.9  (33.0 – 54.9)** | – | 306.5  (92.7 – 520.4)* |

Note: CI=confidence interval.

* p<0.05; **p<0.01; ^†^p<0.10
