# Supplementary material for: Cortisol and α-Amylase Secretion Patterns between and within Depressed and Non-Depressed Individuals
Source: PLoS One. 2015 Jul 6;10(7):e0131002. doi: 10.1371/journal.pone.0131002 (PMC4492984; doi:10.1371/journal.pone.0131002)
Supplement: S5 Table — Note: D = depressed match, N = non-depressed match, > = bigger than, < = smaller than. (DOCX) [file pone.0131002.s006.docx]

S5 Table. Matched-pair comparisons of crude cortisol and α-amylase measures

| *ID* | **Cortisol (nmol/l)** | **Alpha-amylase (U/ml)** | **Slope cortisol** | **Ratio α-amylase over cortisol** |
| --- | --- | --- | --- | --- |
|  | **D N** | **D N** | **D N** | **D N** |
| 1 | 2.74 > 1.78 | 107.8 < 161.6 | -2.04 < -0.56 | 39.4 < 90.8 |
| 2 | 2.40 < 2.55 | 78.8 > 69.0 | -1.66 > -2.06 | 32.8 > 27.1 |
| 3 | 2.53 < 4.79 | 153.1 > 127.4 | -2.14 > -4.25 | 60.5 > 26.6 |
| 4 | 4.30 > 2.65 | 656.7 > 222.0 | -1.47 > -1.51 | 152.7 > 83.8 |
| 5 | 4.99 < 5.28 | 282.4 < 378.5 | -3.36 > -5.02 | 56.6 < 71.7 |
| 6 | 4.80 < 7.22 | 579.3 > 105.3 | -2.79 > -4.47 | 120.7 > 14.6 |
| 7 | 3.39 > 3.16 | 357.0 > 52.3 | -2.38 > -2.81 | 105.3 > 16.6 |
| 8 | 4.49 > 3.11 | 51.8 < 103.5 | -4.12 < -1.53 | 11.5 < 33.3 |
| 9 | 3.14 > 2.97 | 99.3 < 156.5 | -2.00 > -2.29 | 31.6 < 52.7 |
| 10 | 6.40 > 4.14 | 53.6 < 96.8 | -5.88 < -3.14 | 8.4 < 23.4 |
| 11 | 2.28 < 3.86 | 180.7 < 414.3 | -1.88 > -2.94 | 79.2 < 107.3 |
| 12 | 5.00 > 2.62 | 234.7 < 304.4 | -1.67 > -2.17 | 46.9 < 116.2 |
| 13 | 5.18 > 3.14 | 193.3 < 275.2 | -3.52 < -2.04 | 37.3 < 87.7 |
| 14 | 4.68 > 2.52 | 143.7 > 130.1 | -3.32 < -1.83 | 30.7 < 51.6 |
| 15 | 2.46 < 2.69 | 189.6 > 11.7 | -2.14 < -1.75 | 77.1 > 4.4 |
| **Total** | **60% >** | **47% >** | **40% <** | **40% >** |

Note: D=depressed match, N= non-depressed match, >= bigger than, <=smaller than.
