# Supplementary material for: Cortisol and α-Amylase Secretion Patterns between and within Depressed and Non-Depressed Individuals
Source: PLoS One. 2015 Jul 6;10(7):e0131002. doi: 10.1371/journal.pone.0131002 (PMC4492984; doi:10.1371/journal.pone.0131002)
Supplement: S6 Table — D = depressed match, N = non-depressed match, c = controlled for lifestyle factors, > = bigger than, < = smaller than. (DOCX) [file pone.0131002.s007.docx]

S6 Table. Matched-pair comparisons of adjusted cortisol and α-amylase measures

| *ID* | **Cortisol (nmol/l)** | **Alpha-amylase (U/ml)** | **Slope cortisol** | **Ratio α-amylase over cortisol** |
| --- | --- | --- | --- | --- |
|  | **D N** | **D N** | **D N** | **D N** |
| 1c | 3.05 > 1.70 | 86.1 < 161.7 | -2.22 < -0.39 | 28.2 < 95.1 |
| 2c | 2.24 < 3.28 | 79.6 > 69.8 | -1.93 < -1.66 | 35.6 > 21.3 |
| 3c | 2.74 < 3.40 | 159.4 < 164.6 | -2.29 > -4.01 | 58.2 > 48.4 |
| 4c | 4.55 > 2.92 | 629.6 > 290.0 | -2.09 < -1.69 | 138.4 > 99.3 |
| 5c | 4.32 < 5.39 | 275.3 < 418.4 | -3.74 > -5.19 | 63.7 < 77.6 |
| 6c | 4.68 < 6.58 | 558.9 > 99.3 | -2.55 > -4.93 | 119.4 > 15.1 |
| 7c | 3.14 > 3.07 | 356.4 > 56.9 | -1.92 > -2.90 | 113.5 > 18.5 |
| 8c | 4.11 > 3.01 | 53.7 < 92.5 | -4.23 < -1.54 | 13.1 < 30.7 |
| 9c | 3.23 < 3.59 | 114.0 > 108.9 | -1.93 > -2.36 | 35.3 > 30.3 |
| 10c | 4.61 > 4.17 | 50.3 < 87.6 | -6.22 < -3.35 | 10.9 < 21.0 |
| 11c | 2.89 < 4.00 | 171.7 < 362.5 | -1.68 > -2.80 | 59.4 < 90.6 |
| 12c | 6.04 > 2.46 | 213.1 < 278.0 | -3.22 < -1.95 | 35.3 < 113.4 |
| 13c | 5.74 > 3.96 | 197.4 < 312.9 | -3.74 < -1.82 | 34.4 < 79.0 |
| 14c | 4.20 < 4.22 | 109.0 < 139.8 | -3.77 < -1.58 | 26.0 < 33.1 |
| 15c | 2.17 < 2.61 | 212.9 > 11.1 | -2.09 < -1.75 | 98.1 > 4.3 |
| **Total c** | **47% >** | **40% >** | **60% <** | **40% >** |

Note: D=depressed match, N= non-depressed match, c= controlled for lifestyle factors, >= bigger than, <=smaller than.
