# Supplementary material for: Cortisol and α-Amylase Secretion Patterns between and within Depressed and Non-Depressed Individuals
Source: PLoS One. 2015 Jul 6;10(7):e0131002. doi: 10.1371/journal.pone.0131002 (PMC4492984; doi:10.1371/journal.pone.0131002)
Supplement: S7 Table — Note: CI = confidence interval. BDI = Beck Depression Inventory. * p<0.05; **p<0.01; †p<0.10 (DOCX) [file pone.0131002.s008.docx]

**S7 Table. Results of multilevel analysis of the relationship between depression status and crude cortisol and α-amylase, corrected for post-BDI score.**

| **Variables** | **Bootstrapped estimates (95% CI)** | |
| --- | --- | --- |
| *Fixed effects* | **Cortisol (nmol/l)** | **Alpha-amylase (U/ml)** |
| Intercept | 6.54  (6.20 – 8.87)** | 114.5  (97.5 – 131.6)** |
| Depression | 0.13  (-0.24 – 0.50) | 2.8  (-21.8 – 26.1) |
| Post-BDI score | 0.01  (-0.00 – 0.02) | 2.8  (2.1 – 3.6) ** |
| Time | -0.00  (-0.00 – 0.00) | 0.3  (0.2 – 0.5)** |
| Beep afternoon | -3.61  (-3.88 – -3.34)** | 55.2  (43.6 – 66.7)** |
| Beep evening | -5.27  (-5.54 – -5.01)** | 38.9  (27.5 – 50.2)** |

Note: CI=confidence interval. BDI= Beck Depression Inventory.

* p<0.05; **p<0.01; ^†^p<0.10
