# Supplementary material for: Cortisol and α-Amylase Secretion Patterns between and within Depressed and Non-Depressed Individuals
Source: PLoS One. 2015 Jul 6;10(7):e0131002. doi: 10.1371/journal.pone.0131002 (PMC4492984; doi:10.1371/journal.pone.0131002)
Supplement: S8 Table — Note: CI = confidence interval. BMI = Body Mass Index. * p<0.05; **p<0.01; †p<0.10 (DOCX) [file pone.0131002.s009.docx]

**S8 Table. Results of multilevel analysis of the relationship between depression status and crude cortisol and α-amylase measures, corrected for BMI.**

| **Variables** | **Bootstrapped estimates (95% CI)** | | | |
| --- | --- | --- | --- | --- |
| *Fixed effects* | **Cortisol (nmol/l)** | **Alpha-amylase (U/ml)** | **Slope cortisol** | **Ratio α-amylase over cortisol** |
| Intercept | 7.22  (6.66 – 7.78)** | 298.6  (214.5 – 382.8)** | -2.97  (-6.34 – 0.39) ^†^ | 29.5  (7.0 – 52.0)* |
| Depression | 0.35  (0.07 – 0.63)* | 59.4  (37.9 – 80.8)** | -0.18  (-1.34 – 0.97) | 12.6  (3.0 – 22.1)* |
| BMI | -0.03  (-0.05 – -0.01)* | -7.6  (-10.9 – -4.4)** | 0.00  (-0.14 – 0.14) | -0.25  (-1.2 – 0.7) |
| Time | -0.00  (-0.00 – 0.00) | 0.3  (0.1 – 0.5)** | 0.01  (-0.01 – 0.02) | 0.2  (0.0 – 0.3)* |
| Beep afternoon | -3.61  (-3.88 – -3.34)** | 55.5  (44.5 – 66.6)** | – | 58.7  (52.3 – 65.2)** |
| Beep evening | -5.27  (-5.54 – -5.01)** | 43.9  (33.0 – 54.9)** | – | 173.1  (161.8 – 184.5)** |

Note: CI=confidence interval. BMI = Body Mass Index.

* p<0.05; **p<0.01; ^†^p<0.10
