# Supplementary material for: Cortisol and α-Amylase Secretion Patterns between and within Depressed and Non-Depressed Individuals
Source: PLoS One. 2015 Jul 6;10(7):e0131002. doi: 10.1371/journal.pone.0131002 (PMC4492984; doi:10.1371/journal.pone.0131002)
Supplement: S9 Table — Note: CI = confidence interval. BMI = Body Mass Index. * p<0.05; **p<0.01; †p<0.10 (DOCX) [file pone.0131002.s010.docx]

**S9 Table. Results of multilevel analysis of the relationship between depression status and crude cortisol and α-amylase measures, corrected for age.**

| **Variables** | **Bootstrapped estimates (95% CI)** | | | |
| --- | --- | --- | --- | --- |
| *Fixed effects* | **Cortisol (nmol/l)** | **Alpha-amylase (U/ml)** | **Slope cortisol** | **Ratio α-amylase over cortisol** |
| Intercept | 6.60  (6.10 – 7.09)** | 96.4  (43.5 – 149.3)** | -3.72  (-6.88 – -0.56)* | 7.20  (-11.3 – 25.7) |
| Depression | 0.30  (0.23 – 0.57)* | 54.8  (32.3 – 77.2)** | -0.19  (-1.26 – 0.87) | 11.2  (1.7– 30.6)* |
| Age | -0.00  (-0.01 – 0.01) | 0.8  (-0.6 – 2.2) | 0.23  (-0.06 – 0.11) | 0.5  (-0.0 – 0.1) |
| Time | -0.00  (-0.00 – 0.00) | 0.3  (0.1 – 0.5)** | 0.01  (-0.01 – 0.02) | 0.2  (0.0 – 0.3)* |
| Beep afternoon | -3.61  (-3.88 – -3.34)** | 55.5  (44.5 – 66.6)** | – | 58.7  (52.2 – 65.2)** |
| Beep evening | -5.27  (-5.54 – -5.01)** | 43.9  (33.0 – 54.9)** | – | 173.1  (161.7 – 184.5)** |

Note: CI=confidence interval. BMI = Body Mass Index.

* p<0.05; **p<0.01; ^†^p<0.10
